# Atrial high-rate episodes intensify R_2_CHA_2_DS_2_-VASc score for prognostic stratification in pacemaker patients

**DOI:** 10.1038/s41598-023-34784-7

**Published:** 2023-05-11

**Authors:** Yi-Pan Li, Ju-Yi Chen, Tse-Wei Chen, Wei-Da Lu

**Affiliations:** Division of Cardiology, Department of Internal Medicine, National Cheng Kung University Hospital, College of Medicine, National Cheng Kung University, 138 Sheng-Li Road, Tainan, 704 Taiwan

**Keywords:** Cardiology, Medical research, Risk factors

## Abstract

Patients with device detected atrial high-rate episodes (AHRE) have an increased risk of MACE. The R_2_CHA_2_DS_2_-VASc, CHADS_2_, R_2_CHADS_2_ and CHA_2_DS_2_-VASc score have been investigated for predicting major adverse cardiovascular events (MACE) in different groups of patients. We aimed to evaluate the R_2_CHA_2_DS_2_-VASc score in combination with AHRE ≥ 6 min for predicting MACE in patients with dual-chamber PPM but no prior atrial fibrillation (AF). We retrospectively enrolled 376 consecutive patients undergoing dual-chamber PPM implantation and no prior AF. The primary endpoint was subsequent MACE. For all patients in the cohort, CHADS_2_, R_2_CHADS_2_, CHA_2_DS_2_-VASc, R_2_CHA_2_DS_2_-VASc scores and AHRE ≥ or < 6 min were determined. AHRE was recorded as a heart rate > 175 bpm (Medtronic) or > 200 bpm (Biotronik) lasting ≥ 30 s. Multivariate Cox regression analysis with time-dependent covariates was used to determine the independent predictors of MACE. ROC-AUC analysis was performed for CHADS_2_, R_2_CHADS_2_, CHA_2_DS_2_-VASc, and R_2_CHA_2_DS_2_-VASc scores and then adding AHRE ≥ 6 min to the four scores. The median age was 77 years, and 107 patients (28.5%) developed AHRE ≥ 6 min. After a median follow-up of 32 months, 46 (12.2%) MACE occurred. Multivariate Cox regression analysis showed that R_2_CHA_2_DS_2_-VASc score (HR, 1.485; 95% CI, 1.212–1.818; *p* < 0.001) and AHRE ≥ 6 min (HR, 2.125; 95% CI, 1.162–3.887; *p* = 0.014) were independent predictors for MACE. The optimal R_2_CHA_2_DS_2_-VASc score cutoff value was 4.5 (set at ≥ 5), with the highest Youden index (AUC, 0.770; 95% CI, 0.709–0.831; *p* < 0.001). ROC-AUC analysis of the four risk scores separately combined with AHRE ≥ 6 min all showed better discriminatory power than the four scores alone (All *Z*-statistic *p* < 0.05). In patients with PPM who develop AHRE ≥ 6 min, it is crucial to perform risk assessment with either four scores to further stratify risk for MACE.

## Introduction

The increased use of cardiac implantable electronic device (CIED) such as dual chamber permanent pacemakers (PPM) or internal cardiac defibrillators (ICDs) can detect episodes of atrial tachyarrhythmias, including atrial tachycardia, atrial flutter and atrial fibrillation (AF) in patients with an atrial lead. These tachycardia episodes, commonly asymptomatic, are known as atrial high-rate episodes (AHRE), also called subclinical AF^1,2^. The rate criterion for AHRE varies in different studies and is > 175 bpm on current guideline, and there is a wide range of duration cut-offs, from 10 to 20 s to > 24 h^1^. AHRE were reported in 10–35% in studies including patients without known AF^2,3^. Previous studies have reported that AHRE ≥ 5–6 min increase the risk of clinical AF^4^ and ischemic stroke^3,4^. The increased risk of major adverse cardiovascular events (MACE), especially myocardial infarction has also been reported in patients with AF^5,6^ but only few studies in those with AHRE. The recent studies have demonstrated AHRE are associated with increased risk of cardiovascular death^7^, and MACE^8^. A higher burden leads to a higher risk of thrombo-embolism, heart failure and MACE^8–10^, indicating that dual chamber PPMs should be interrogated regularly to identify AHRE, and these patients should undergo further risk assessment for MACE.

CHADS_2_ and CHA_2_DS_2_-VASc scores are used for stroke risk stratification in patients with AF and the risk scores decide the subsequent use of oral anticoagulant according to current guideline^1^. R_2_CHADS_2_ and R_2_CHA_2_DS_2_-VASc scoring system adding 2 points for renal dysfunction to CHADS_2_ and CHA_2_DS_2_-VASc scores had a better performance to stratify thromboembolic risk in patients with AF^11,12^. These scores are composed of parameters which are known as atherosclerotic risk factors for cardiovascular events. In addition to risk assessment for stroke, these scores have been proposed to predict cardiovascular mortality and all-cause death in different sets of population, including patients with high cardiovascular risk^13^, chest pain^14^, coronary artery disease^15^, acute coronary syndrome^12,16–19^, heart failure^20^, sick sinus syndrome^21^ and patients undergoing transcatheter aortic valve replacement^22^. The predictive ability of the R_2_CHA_2_DS_2_-VASc score on MACE occurrence in patients with dual chamber PPMs and no prior AF and the combination of AHRE ≥ 6 min and R_2_CHA_2_DS_2_-VASc score have not yet been studied.

Accordingly, we investigated the performance of R_2_CHA_2_DS_2_-VASc score in comparison to other risk scales, including CHADS_2_, CHA_2_DS_2_-VASc and R_2_CHADS_2_ scores, and also elucidated the predictive value of AHRE ≥ 6 min in combination with R_2_CHA_2_DS_2_-VASc score and other scores for MACE occurrence. The novelty of this study is that there is no previous research to examine the discriminating ability of R_2_CHA_2_DS_2_-VASc and the other three scores in combination with AHRE ≥ 6 min to predict MACE.

## Methods

Consecutive patients ≥ 18 years of age who underwent dual-chamber PPM implantation (Medtronic, Minneapolis, MN, USA or Biotronik, Berlin, Germany) in the Cardiology Department of National Cheng Kung University Hospital from January 2014 to April 2021 were retrospectively included.

### Ethical considerations

The protocol for this cohort study was reviewed and approved by the ethics committee of National Cheng Kung University Hospital and conducted according to the guidelines of the International Conference on Harmonization for Good Clinical Practice (B-ER-108–278).

### Data collection and definitions^23^

Patients’ medical histories and data regarding comorbidities and echocardiographic parameters were collected from medical records for retrospective evaluation. Diabetes mellitus was defined as the presence of symptoms and random plasma glucose concentration ≥ 200 mg/dL, fasting plasma glucose concentration ≥ 126 mg/dL, 2-h plasma glucose concentration ≥ 200 mg/dL from a 75-g oral glucose tolerance test, or use of medication for diabetes mellitus. Hypertension was defined as in-office systolic blood pressure ≥ 140 mmHg and/or diastolic blood pressure ≥ 90 mmHg or use of antihypertensive medication. Dyslipidemia was defined as low-density lipoprotein ≥ 140 mg/dL, high-density lipoprotein < 40 mg/dL, triglycerides ≥ 150 mg/dL, or use of medication for dyslipidemia. Chronic kidney disease (CKD) was defined as an estimated glomerular filtration rate (eGFR) < 60 mL/min/1.73 m^2^ for at least 3 months^23^. Previous AF was defined as any documented AF on 12-lead electrocardiography (ECG) or Holter recordings ≥ 30 s before the date of implantation of PPM. The primary endpoint for this study was the occurrence of MACE after the date of PPM implantation, including ST elevation myocardial infarction (MI), non-ST elevation MI, unstable angina, or death (cardiac or noncardiac). We excluded stroke from the components of MACE because the CHA_2_DS_2_-VASc and R_2_CHA_2_DS_2_-VASc scoring systems have been validated to predict the incidence of stroke. For each outcome, only the first event of that outcome in a given patient was included. For the composite outcome, only the first event in a given patient was included.

AHRE was detected by a CIED as a heart rate > 175 bpm (Medtronic) or > 200 bpm (Biotronik) and at least 30 s of atrial tachyarrhythmia recorded by the devices on any day during the study periods^23^. Atrial sensitivity was programmed to 0.3 mV with bipolar sensing of Medtronic devices and 0.2 mV with bipolar sensing of Biotronik devices. AHRE electrograms extracted from the devices via telemetry at each office visit (every 3–6 months) were reviewed by at least one experienced electrophysiologist, who considered the possibility that AHRE included lead noise or artifacts, far-field R-waves, or paroxysmal supraventricular tachycardia and who visually confirmed AF in the detected AHRE. The duration of detected AHRE was recorded and we divided the patients into two groups according to whether detected AHRE duration was more than 6 min or not.

### Scoring system assessments

The CHADS_2_ score^1^ ranges from 0 to 6. A history of heart failure, hypertension, or diabetes mellitus and age ≥ 75 years are calculated as 1 point; prior stroke, transient ischemic attack (TIA) or thromboembolism are each calculated as 2 points. The CHA_2_DS_2_-Vasc score^1^ ranges from 0 to 9. Patients are given 1 point for history of heart failure, hypertension, diabetes, or vascular disease; age 65–74 years; and female sex and 2 points for age ≥ 75 years and prior stroke, transient ischemic attack, or systemic thromboembolism. The R_2_CHADS_2_ score^11^ ranges from 0 to 8, in which 1 point is assigned for a history of heart failure, hypertension, or diabetes mellitus and age ≥ 75 years, and 2 points are assigned for prior stroke, transient ischemic attack, or systemic thromboembolism and chronic kidney disease (CKD), which is defined as an estimated glomerular filtration rate (eGFR) < 60 mL/min/1.73 m^2^ for at least 3 months.

The R_2_CHA_2_DS_2_-VASc score ranges from 0 to 11, in which 1 point is assigned for history of heart failure, hypertension, diabetes, or vascular disease; age 65–74 years, and female sex, and 2 points are assigned for CKD, age ≥ 75 years and prior stroke, transient ischemic attack, or systemic thromboembolism.

### Statistical analysis

Categorical variables are presented as percentages, and continuous variables are presented as the mean and standard deviation for normally distributed values or as the median and interquartile interval (IQI) for nonnormally distributed values. Normal distribution of continuous variables was assessed using the Kolmogorov–Smirnov method. Pearson’s chi-square test or Fisher's exact test was used to determine differences in baseline characteristics for categorical variables and differences between the risk scores groups. A two-sample Student’s t test or the Mann–Whitney U test was used to analyze continuous variables. Survival was estimated using the Kaplan–Meier method, and differences in survival were evaluated using the log-rank test. Multivariate Cox regression analysis with time-dependent covariate was used to identify variables associated with MACE occurrence, reported as hazard ratios (HR) with 95% confidence intervals (CI). Parameters with a *p* < value 0.05 in the univariate analysis were entered into multivariate analysis, but variables already considered by the R_2_CHA_2_DS_2_-VASc score were not evaluated separately in any multivariate Cox regression analysis regardless of their significance in the univariate analysis. Previous studies have used Cox regression analysis to investigate the relationship of AHRE and stroke, CV mortality and MACE^3,7,8^. The receiver-operating characteristic (ROC) area under the curve (AUC) of the CHA_2_DS_2_-VASc, CHADS_2_, R_2_CHADS_2_, R_2_CHA_2_DS_2_-VASc score, and the associated 95% confidence intervals (CI) were evaluated for association with future MACE after PPM implantation. The optimal cutoff values with the highest Youden index were chosen based on the results of ROC analysis and used to evaluate the associated values of the R_2_CHA_2_DS_2_-VASc score for determining MACE. To further examine discriminatory power of the risk assessment model, we performed ROC-AUC analysis of combined AHRE and the risk scores. We used DeLong test^24^, a nonparametric approach to the comparison of the area under two or more ROC curves, to compare the performances of the four scores and different models. For all comparisons, *p* < 0.05 was considered statistically significant. All data were analyzed using SPSS statistical package version 23.0 (SPSS Inc. Chicago, IL, USA).

### Ethical approval

Approved by the Institutional Review Board of National Cheng Kung University Hospital (B-ER-108–278).

## Results

Between January 1, 2014, and April 2021, a total of 511 consecutive patients who underwent dual-chamber PPM implantation at National Cheng Kung University Hospital were recruited. Patients with previous AF (n = 135) were excluded. The final analysis included 376 patients, of whom 46 (12.2%) had experienced MACE.

The median follow-up period was 32 months after dual-chamber PPM implantation. Table 1 shows the patients’ baseline demographic and clinical characteristics according to the presence or absence of MACE. The median age was 77 (69–84) years, and 56.1% of participants were male. The median BMI was 24.8 kg/m^2^, and most patients were not obese. The brands of PPMs were Medtronic (58.5%) and Biotronik (41.5%). The most common indication for PPM implantation was sinus node dysfunction (66.0%), followed by atrioventricular block (34.0%) (Table 1). The overall median percentages of atrial pacing and ventricular pacing were 41.1% and 7.1%, respectively. High percentages of diabetes (50.5%), hypertension (91%), and dyslipidemia (87.5%) suggested a relatively high risk of MACE for the entire study cohort. One hundred thirty-seven patients (36.4%) used antiplatelet therapy, 99 patients (26.3%) took beta-blockers, 146 patients (38.9%) took RAAS inhibitors, and 131 patients (34.8%) took statins at baseline. The total number of MACE was 46 (12.2%). The median R_2_CHA_2_DS_2_-VASc score was 4 (range, 3–6), the median CHA_2_DS_2_-VASc score was 3 (range, 2–4), the median CHADS_2_ score was 3 (range, 2–3), and the median R_2_CHADS_2_ score was 3 (range, 2–5). One hundred seven (28.5%) patients had AHRE ≥ 6 min. Twenty five of the 107 (23.3%) patients with AHRE ≥ 6 min had MACE. In those without AHRE ≥ 6 min, 21 of 269 (7.8%) patients had MACE, which was significantly lower (*p* < 0.05).

**Table 1:** Baseline characteristics of the overall study group and with/without major cardiovascular events.

	All patients (N = 376)	Major cardiovascular events		Univariate *p*	Multivariate Cox regression analysis		
		Yes (N = 46)	No (N = 330)		HR	95% CI	p
Age (years)	77 (69–84)	77 (71–83)	77 (68–85)	0.710			
Sex				0.050			
Male	211 (56.1%)	32 (69.6%)	179 (54.2%)				
Female	165 (43.9%)	14 (30.4%)	151 (45.8%)				
BMI (kg/m^2^)	24.8 (22.9–25.9)	25.4 (23.3–26.2)	24.6 (22.6–25.9)	0.244			
Device type				0.978			
Medtronic	220 (58.5%)	27 (58.7%)	193 (58.5%)				
Biotronik	156 (41.5%)	19 (41.3%)	137 (41.5%)				
Primary indication				0.275			
Sinus node dysfunction	248 (66.0%)	35 (76.1%)	213 (64.5%)				
Atrioventricular block	128 (34.0%)	11 (23.9%)	117 (35.5%)				
Atrial pacing (%)	41.1 (13.4–80.5)	35.2 (4.8–72.5)	43.9 (14.3–80.6)	0.225			
Ventricular pacing (%)	7.1 (0.2–96.3)	19.8 (0.2–73.5)	7.1 (0.2–96.8)	0.888			
Hypertension	342 (91.0%)	45 (97.8%)	297 (90.0%)	0.100			
Diabetes mellitus	190 (50.5%)	38 (82.6%)	152 (46.1%)	< 0.001			
Dyslipidemia	329 (87.5%)	44 (95.7%)	285 (86.4%)	0.094			
Chronic obstructive pulmonary disease	18 (4.8%)	3 (6.5%)	15 (4.5%)	0.472			
Prior myocardial infarction	73 (19.4%)	24 (52.2%)	49 (14.8%)	< 0.001			
Prior stoke	16 (4.3%)	2 (4.3%)	14 (4.2%)	1.000			
Heart failure				< 0.001			
Preserved LVEF	30 (8.0%)	6 (13.0%)	24 (7.3%)				
Reduced LVEF	41 (10.9%)	20 (43.5%)	21 (6.4%)				
None	305 (81.1%)	20 (43.5%)	285 (86.4%)				
Chronic kidney disease	143 (38.0%)	30 (65.2%)	113 (34.2%)	< 0.001			
Hyperthyroidism	10 (2.7%)	1 (2.2%)	9 (2.7%)	1.000			
Hypothyroidism	12 (3.2%)	2 (4.3%)	10 (3.0%)	0.647			
Echo parameters							
LVEF (%)	69.0 (61.0–74.6)	55.5 (40.0–74.0)	69.5 (62.0–75.0)	0.001	0.979	0.958–1.001	0.058
Mitral E/e’	11.4 (9.0–14.0)	12.0 (10.0–16.0)	11.0 (9.0–14.0)	0.045	0.980	0.928–1.034	0.458
LA diameter (cm)	3.7 (3.2–4.0)	4.0 (3.6–4.3)	3.6 (3.2–4.0)	< 0.001	1.550	0.933–2.572	0.090
RV systolic function (s’, m/s)	12.0 (12.0–14.0)	11.8 (10.0–12.0)	12.0 (12.0–14.0)	< 0.001	0.825	0.679–1.003	0.054
Drugs prescribed at baseline							
Antiplatelets	137 (36.4%)	38 (82.6%)	99 (30.0%)	< 0.001			
Anticoagulants	34 (9.0%)	5 (10.9%)	29 (8.8%)	0.645			
Beta blockers	99 (26.3%)	26 (56.5%)	73 (22.1%)	< 0.001			
Amiodarone	46 (12.2%)	12 (26.1%)	34 (10.3%)	0.002			
Non-DHP CCBs	14 (3.7%)	2 (4.3%)	12 (3.6%)	0.684			
RAAS inhibitors	146 (38.9%)	23 (50.0%)	123 (37.4%)	0.100			
Diuretics	60 (16.0%)	16 (34.8%)	44 (13.3%)	< 0.001			
Statins	131 (34.8%)	23 (50.0%)	108 (32.7%)	0.021			
SGLT2 inhibitors	5 (1.3%)	1 (2.2%)	4 (1.2%)	0.481			
CHA_2_DS_2_-VASc score	3 (2–4)	4 (4–5)	3 (2–4)	< 0.001			
CHADS_2_ score	2 (2–3)	3 (3–4)	2 (1–3)	< 0.001			
R_2_CHA_2_DS_2_-VASc score	4 (3–6)	6 (5–7)	4 (2–5)	< 0.001	1.485	1.212–1.818	< 0.001
R_2_CHADS_2_ score	3 (2–5)	5 (3–6)	3 (2–4)	< 0.001			
AHRE ≥ 6 min	107 (28.5%)	25 (54.3%)	82 (24.8%)	< 0.001	2.125	1.162–3.887	0.014

Data are presented as the median (interquartile interval) or n (%). Nonparametric continuous variables, as assessed using the Kolmogorov–Smirnov method, were analyzed using the Mann–Whitney U test. Statistical significance was set at *p* < 0.05.

BMI, body mass index; PM, pacemaker; LVEF, left ventricular ejection fraction; LA, left atrium; RV, right ventricle; non-DHP CCBs, nondihydropyridine calcium channel blockers; RAAS, renin–angiotensin–aldosterone system; SGLT2, sodium glucose cotransporter 2; CHA_2_DS_2_-Vasc score: Range from 0 to 9. History of heart failure, hypertension, diabetes, and vascular disease; age 65–74 years; and female sex are each calculated as 1 point; 75 years or older and prior stroke, TIA, or thromboembolism are each calculated as 2 points; AHRE, atrial high-rate episodes.

### Univariate analysis and multivariate Cox regression analysis to identify independent predictors of MACE

Univariate analysis revealed that MACE occurrence was significantly associated with a history of diabetes mellitus, prior myocardial infarction, heart failure, chronic kidney disease, worse LVEF, higher mitral E/e’, larger left atrial (LA) diameter, worse RV systolic function, higher CHA_2_DS_2_-VASc, CHADS_2_, R_2_CHA_2_DS_2_-VASc, R_2_CHADS_2_ score and AHRE ≥ 6 min (Table 1). In the multivariate Cox regression analysis, we did not include components of the R_2_CHA_2_DS_2_-VASc score such as chronic kidney disease, heart failure, diabetes mellitus, and prior myocardial infarction. The R_2_CHA_2_DS_2_-VASc score was an independent predictor of MACE in multivariate Cox regression analysis (HR, 1.485; 95% CI, 1.212–1.818; p < 0.001). AHRE ≥ 6 min was another stronger independent predictor of MACE occurrence (HR, 2.125; 95% CI, 1.162–3.887; p = 0.014). A larger LA diameter was associated with a trend toward increased MACE occurrence (HR, 1.550; 95% CI, 0.933–2.572, p = 0.090).

### ROC-AUC determination of R_2_CHA_2_DS_2_-VASc score cutoff values for factors predictive of future MACE and survival analysis

**Figure 1:**
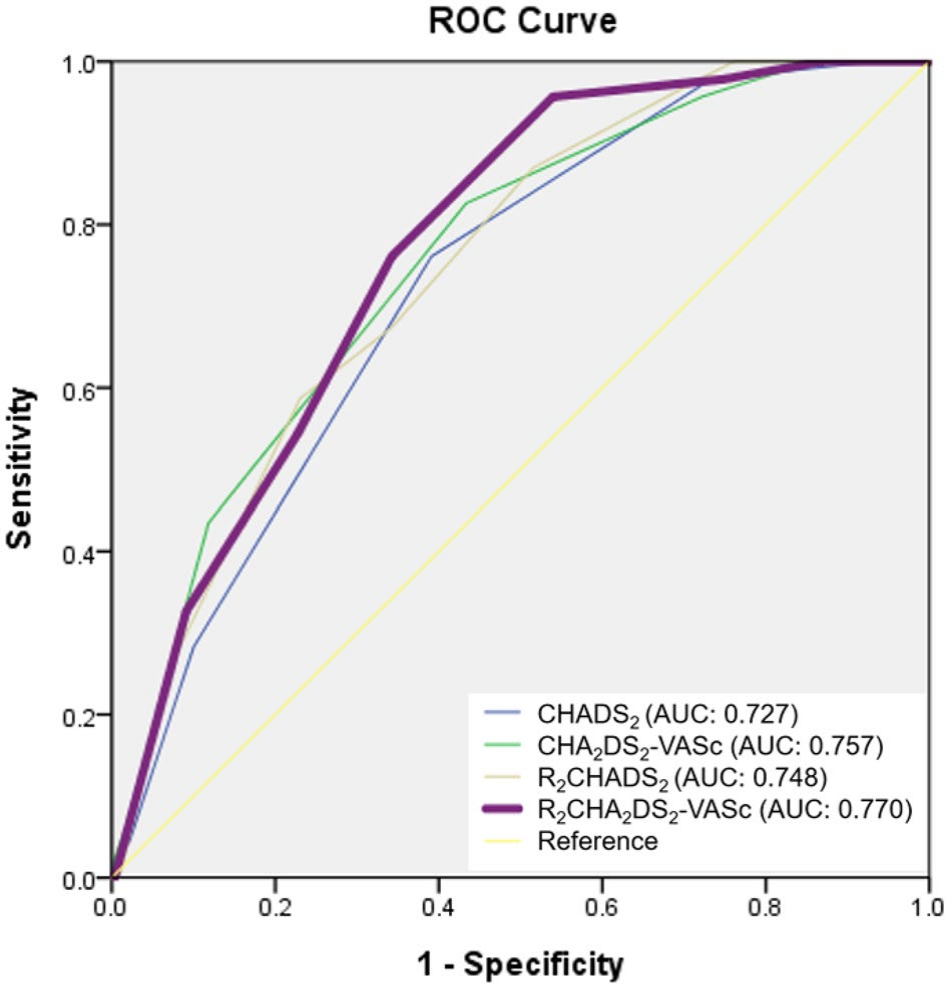
Receiver operating characteristic analysis of the R_2_CHA_2_DS_2_-VASc score in patients with permanent pacemakers with subsequent major cardiovascular events. The R_2_CHA_2_DS_2_-VASc score: optimal cutoff value with the highest Youden index, 4.5; sensitivity, 76.1%; specificity, 65.8%; AUC, 0.770; 95% CI, 0.709–0.831; *p* < 0.001. The other C-statistics: CHA_2_DS_2_-VASc score 0.757, 95% CI = 0.688–0.826, *p* < 0.001; R_2_CHADS_2_ score 0.748, 95% CI = 0.681–0.815, *p* < 0.001; CHADS_2_ score 0.727, 95% CI = 0.659–0.796, *p* < 0.001. There are no statistically significant differences in the C-indexes among the four scores.

The ROC analysis of the R_2_CHA_2_DS_2_-VASc score showed that the optimal cutoff value for predicting the occurrence of MACE was 4.5 according to the highest Youden index (sensitivity, 76.1%; specificity, 65.8%; AUC, 0.770; 95% CI, 0.709–0.831; *p* < 0.001; Fig. 1). In practice, the cutoff value would be set at ≥ 5. The other AUC values were as follows: CHA_2_DS_2_-VASc score: 0.757, 95% CI = 0.688–0.826, *p* < 0.001; R_2_CHADS_2_ score: 0.748, 95% CI = 0.681–0.815, *p* < 0.001; and CHADS_2_ score: 0.727, 95% CI = 0.659–0.796, *p* < 0.001, which represented acceptable discriminating ability of MACE prediction. We compared AUC values of each two different risk scores using *Z*-statistic. The corresponding comparisons were as follows: CHADS_2_ versus R_2_CHADS_2_ (*Z*-statistic: 0.909, *p* > 0.05), CHADS_2_ versus CHA_2_DS_2_-VASc (*Z*-statistic: 0.799, *p* > 0.05), CHADS_2_ versus R_2_CHA_2_DS_2_-VASc (*Z*-statistic: 1.284, *p* > 0.05), R_2_CHADS_2_ versus R_2_CHA_2_DS_2_-VASc (*Z*-statistic: 0.965, *p* > 0.05), R_2_CHADS_2_ versus CHA_2_DS_2_-VASc (*Z*-statistic: 0.22, *p* > 0.05), and CHA_2_DS_2_-VASc versus R_2_CHA_2_DS_2_-VASc (*Z*-statistic: 0.537, *p* > 0.05), demonstrating that there was no statistically significant differences in the AUC values among the four scores. The event numbers and rates of the four scores were listed in Table 2.

**Table 2:** The event rates of each risk score.

CHADS_2_	0	1	2	3	4	5	6					
Number	21	68	123	118	37	7	2					
Events	0	1	10	22	11	1	1					
MACE(%)	0%	1.5%	8.1%	18.6%	29.7%	14.3%	50%					
R_2_CHADS_2_	0	1	2	3	4	5	6	7	8			
Number	21	58	87	66	41	67	33	0	0			
Events	0	0	6	9	4	15	12	0	0			
MACE(%)	0%	0%	6.9%	13.6%	9.8%	22.4%	36.4%	0%	0%			
CHA_2_DS_2_-VASc	0	1	2	3	4	5	6	7	8	9		
Number	11	36	47	101	122	50	7	2	0	0		
Events	0	0	2	6	18	17	2	1	0	0		
MACE(%)	0%	0%	4.3%	5.9%	14.8%	34.3%	28.6%	50%	0%	0%		
R_2_CHA_2_DS_2_-VASc	0	1	2	3	4	5	6	7	8	9	10	11
Number	11	32	41	70	74	48	55	38	6	1	0	0
Events	0	0	1	1	9	10	10	13	2	0	0	0
MACE(%)	0%	0%	2.4%	1.4%	12.2%	20.8%	18.2%	34.2%	33.3%	0%	0%	0%

Preliminarily, we divided our patients into three risk groups according to the R_2_CHA_2_DS_2_-VASc score: low risk (R_2_CHA_2_DS_2_-VASc score: 0–1), intermediate risk (score: 2–4) and high risk (score: 5–11). The MACE rates were 0%, 5.9% and 23.6%, respectively.

**Figure 2:**
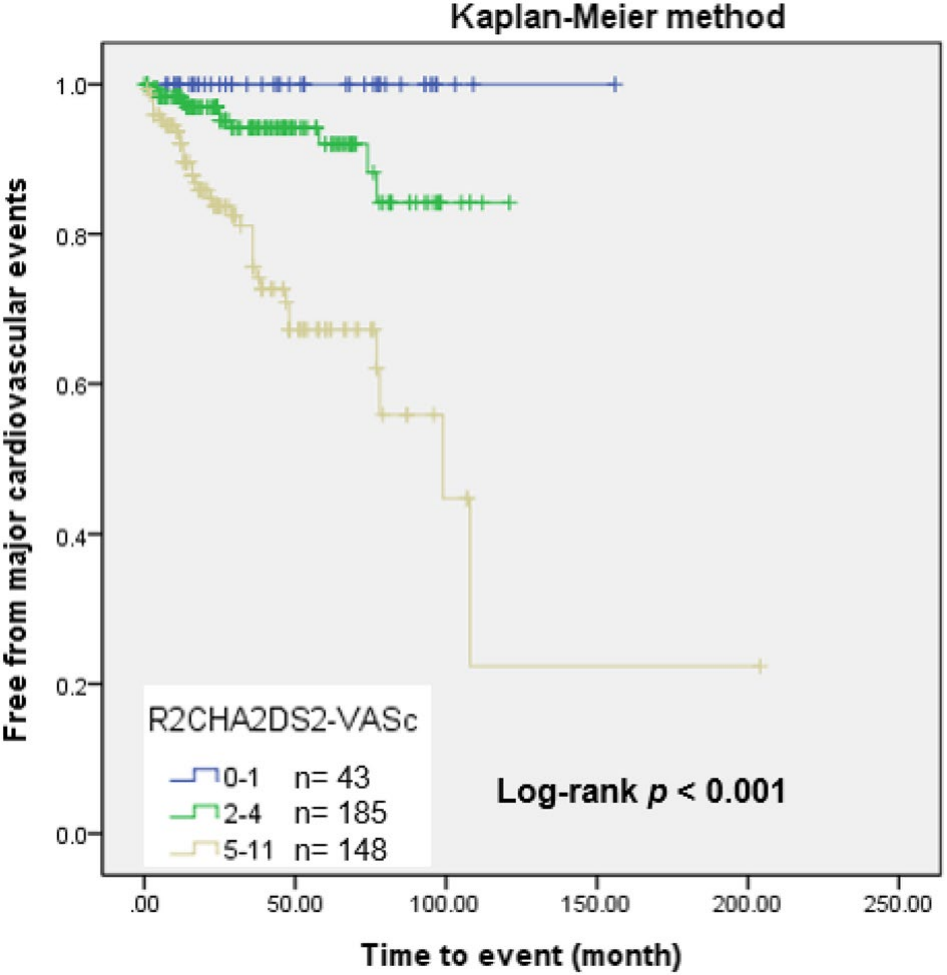
Kaplan–Meier curves depicting the cumulative survival rates free from major cardiovascular events with respect to the R_2_CHA_2_DS_2_-VASc score, divided into three categories according to MACE rates: low risk (0–1; 0%), intermediate risk (2–4; 5.9%) and high risk (5–11; 23.6%), log-rank *p* < 0.001.

**Figure 3:**
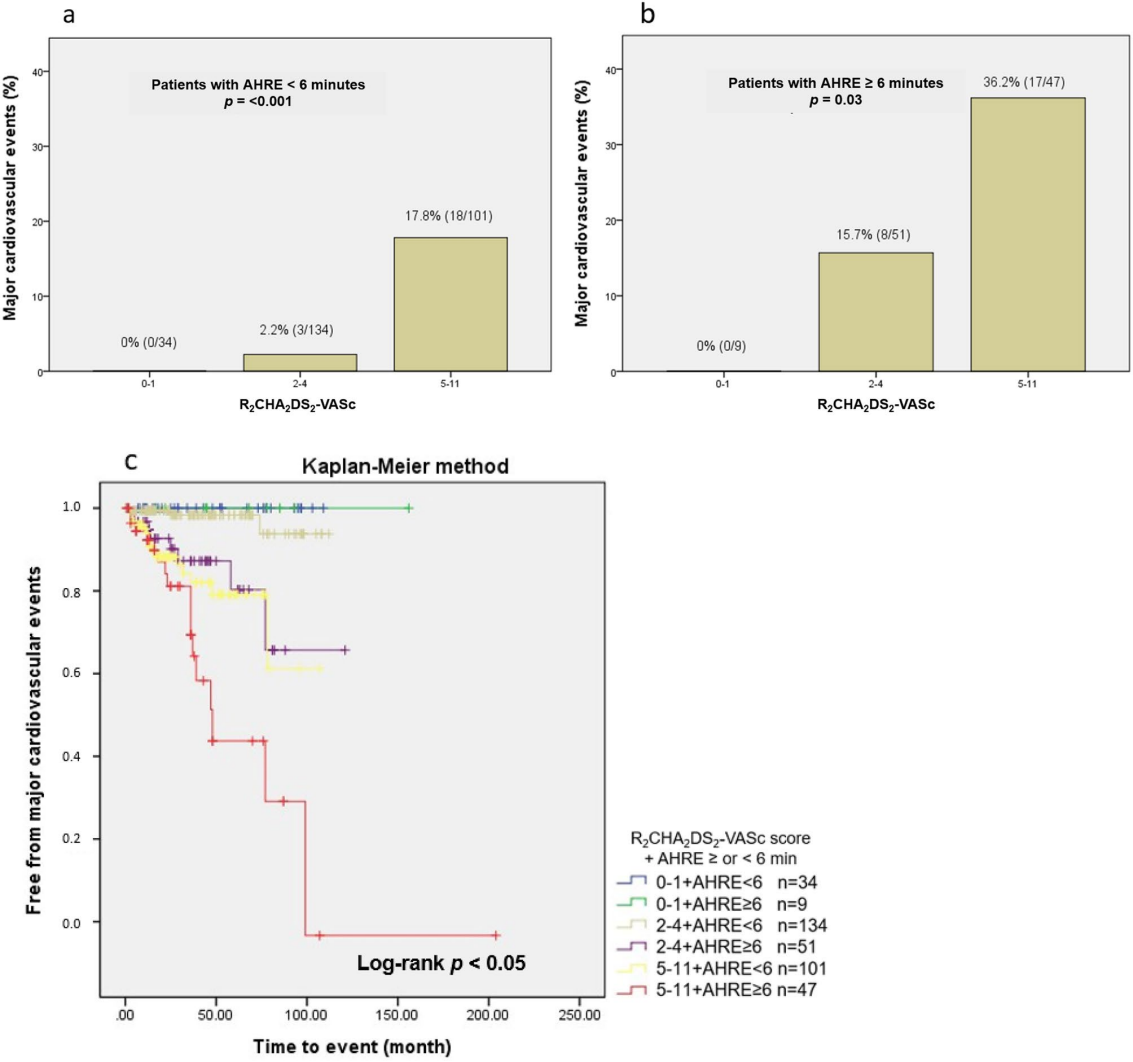
The major cardiovascular event rate significantly increased with increasing R_2_CHA_2_DS_2_-VASc scores, whether in patients with (**A**) AHRE ≥ or (**B**) < 6 min. (**C**) Kaplan–Meier curves depicting the cumulative survival rates free from major cardiovascular events with respect to the R_2_CHA_2_DS_2_-VASc score and AHRE ≥ or < 6 min, log-rank *p* < 0.05.

Kaplan–Meier survival curves showed a significant decrease in cumulative rates of freedom from MACE (log-rank p < 0.001) as the R_2_CHA_2_DS_2_-VASc score increased according to the three risk categories (Fig. 2). In the three groups, we further analyzed the occurrence of MACE in patients with AHRE ≥ or < 6 min (Fig. 3a,b). The MACE rates in patients with AHRE < 6 min were as follows: low risk (0%), intermediate risk (2.2%) and high risk (17.8%) (*p* < 0.001). The MACE rates in patients with AHRE ≥ 6 min were higher: low risk (0%), intermediate (15.7%), and high risk (36.2%) (*p* = 0.013). There were significant differences in MACE occurrence among the three groups regardless of AHRE ≥ 6 or < 6 min. There were also significant differences between the event rates of the intermediate-risk group with AHRE ≥ 6 min (15.7%) and the intermediate-risk group with AHRE < 6 min (2.2%), and between the high-risk group with AHRE ≥ 6 min (36.2%) and the high-risk group with AHRE < 6 min (17.8%), respectively. Kaplan–Meier survival curves showed a significant decrease in cumulative rates of freedom from MACE (log-rank *p* < 0.05) in high-risk group and intermediate-risk group with AHRE ≥ 6 min (Fig. 3c).

### ROC-AUC determination of combined AHRE and the four scores for predicting future MACE

**Figure 4:**
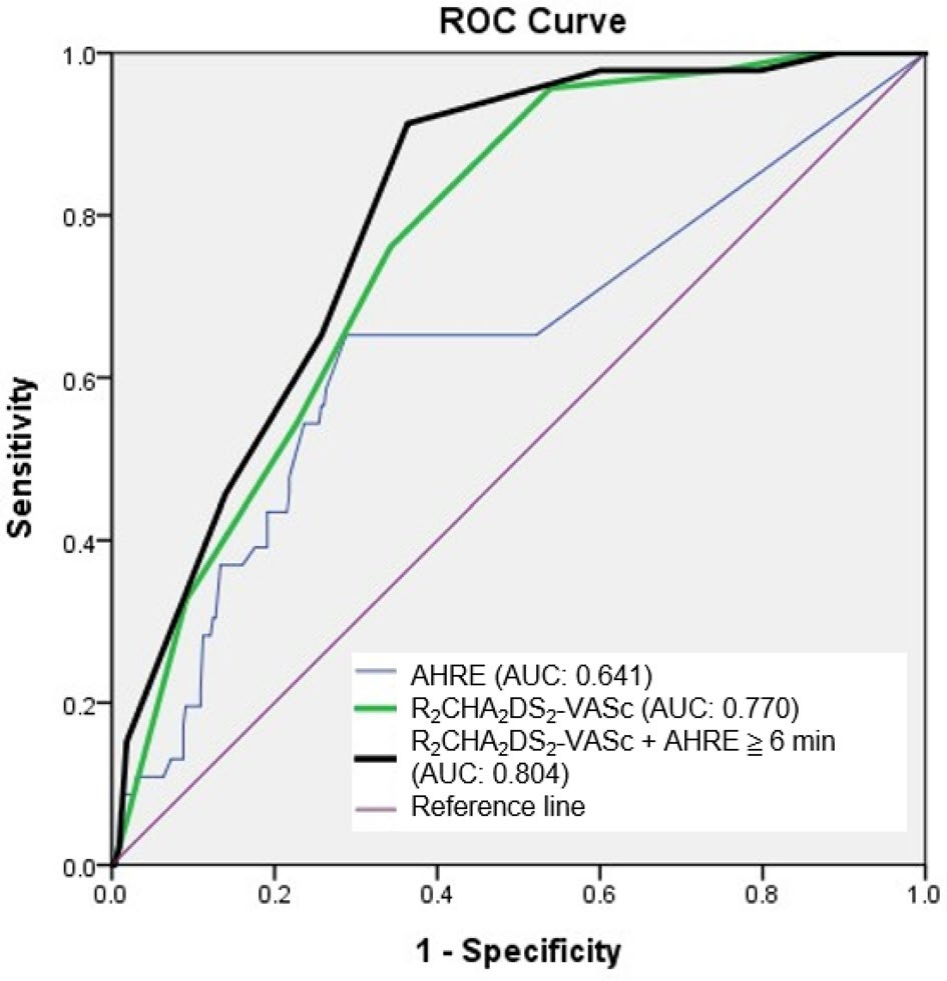
Receiver operating characteristic analysis of the R_2_CHA_2_DS_2_-VASc score in combination with AHRE ≥ 6 min for predicting major cardiovascular adverse events. The AUC values of R_2_CHA_2_DS_2_-VASc score in combination with AHRE ≥ 6 min, R_2_CHA_2_DS_2_-VASc score and AHRE are 0.804 (95% CI = 0.761–0.843, p < 0.001), 0.770 (95% CI = 0.724–0.812, *p* < 0.001) and 0.641 (95% CI = 0.590–0.689, *p* < 0.001). The comparisons of each other are R_2_CHA_2_DS_2_-VASc score in combination with AHRE ≥ 6 min versus R_2_CHA_2_DS_2_-VASc score (*Z*-statistic: 3.265, *p* = 0.0011), R_2_CHA_2_DS_2_-VASc score in combination with AHRE ≥ 6 min versus AHRE (*Z*-statistic: 3.074, *p* = 0.0021), R_2_CHA_2_DS_2_-VASc score versus AHRE (*Z*-statistic: 2.126, *p* = 0.0335).

The patients with intermediate and high risk according to R_2_CHA_2_DS_2_-VASc score had higher risk for MACE rates in the presence of AHRE ≥ 6 min (15.7% vs. 2.2% and 36.2% vs. 17.8%, respectively), indicating that combination of the score and AHRE may enhance the discrimination ability. We performed further ROC analysis of combined AHRE and the four scores for predicting MACE. The results showed significantly higher AUC values as compared to each score alone (Table 3). As listed in Fig. 4, the combined AHRE ≥ 6 min and R_2_CHA_2_DS_2_-VASc score demonstrated higher AUC value (0.804, 95%CI: 0.761–0.843) than R_2_CHA_2_DS_2_-VASc score alone (0.770, 95%CI: 0.724–0.812) (*Z*-statistic = 3.265, *p* = 0.0011) and AHRE (*Z*-statistic = 3.074, *p* = 0.0021), suggestive of better discriminating ability to predict MACE. The risk stratification models, R_2_CHA_2_DS_2_-VASc score plus AHRE ≥ 6 min and CHA_2_DS_2_-VASc score plus AHRE ≥ 6 min had numerically higher AUC values of 0.804 and 0.810, respectively, indicating excellent discrimination. The corresponding comparisons of AUC values of the four combined risk stratification models (CHADS_2_ plus AHRE ≥ 6 min, R_2_CHADS_2_ plus AHRE ≥ 6 min, CHA_2_DS_2_-VASc plus AHRE ≥ 6 min, and R_2_CHA_2_DS_2_-VASc plus AHRE ≥ 6 min) with each other by using DeLong test showed no statistically significant difference between the models (All *p* > 0.05). The event rates of the combination of AHRE for each score were listed in Table 4.

**Table 3:** The ROC-AUC analysis of CHADS_2_, R_2_CHADS_2_, CHA_2_DS_2_-VASc, R_2_CHA_2_DS_2_-VASc score with combination of AHRE ≥ 6 min and each score.

Risk models	AUC values	Compared with	AUC values	*Z*-statistic	*p* value
AHRE ≥ 6 min plus CHADS_2_	0.768 (95% CI = 0.722–0.810, *p* < 0.001)	CHADS_2_	0.727 (95% CI = 0.659–0.796, p < 0.001)	2.366	0.018
AHRE ≥ 6 min plus R_2_CHADS_2_	0.773 (95% CI = 0.727–0.814, *p* < 0.001)	R_2_CHADS_2_	0.748 (95% CI = 0.681–0.815, p < 0.001)	2.313	0.0207
AHRE ≥ 6 min plus CHA_2_DS_2_-VASc	0.810 (95% CI = 0.766–0.848, *p* < 0.001)	CHA_2_DS_2_-VASc	0.757 (95% CI = 0.688–0.826, p < 0.001)	3.126	0.0018
AHRE ≥ 6 min plus R_2_CHA_2_DS_2_-VASc	0.804 (95% CI = 0.761–0.843, *p* < 0.001)	R_2_CHA_2_DS_2_-VASc	0.770 (95% CI, 0.709–0.831; p < 0.001)	3.265	0.0011

**Table 4:** The event rates of combination of AHRE ≥ or < 6 min and each score.

CHADS_2_ score											
	0 n = 21	1 n = 68	2 n = 123	3 n = 118	4 n = 37	5 n = 7	6 n = 2				
AHRE < 6 min n = 269	0% 0/17	1.9% 1/53	8.0% 7/87	7.6% 6/79	22.2% 6/27	16.7% 1/6	0% 0/0				
AHRE ≥ 6 min n = 107	0% 0/4	0% 0/15	8.3% 3/36	41% 16/39	50% 5/10	0% 0/1	50% 1/2				
CHA_2_DS_2_-VASc score											
	0 n = 11	1 n = 36	2 n = 47	3 n = 101	4 n = 122	5 n = 50	6 n = 8	7 n = 2	8 n = 0	9 n = 0	
AHRE < 6 min n = 269	0% 0/9	0% 0/27	3.3% 1/30	2.9% 2/70	6.9% 6/87	24.4% 10/41	25% 1/4	0% 0/1	0% 0/0	0% 0/0	
AHRE ≥ 6 min n = 107	0% 0/2	0% 0/9	5.9% 1/17	12.9% 4/31	34.3% 12/35	77.8% 7/9	33.3% 1/3	0% 0/1	0% 0/0	0% 0/0	
R_2_CHADS_2_ score											
	0 n = 21	1 n = 58	2 n = 87	3 n = 66	4 n = 41	5 n = 67	6 n = 33	7 n = 2	8 n = 1		
AHRE < 6 min n = 269	0% 0/17	0% 0/43	7.9% 5/63	3.8% 2/52	7.1% 2/28	14.6% 6/41	26.1% 6/23	0% 0/2	0% 0/0		
AHRE ≥ 6 min n = 107	0% 0/4	0% 0/15	4.2% 1/24	50% 7/14	15.4% 2/13	34.6% 9/26	60% 6/10	0% 0/0	0% 0/1		
R_2_CHADS_2_-VASc score											
	0 n = 11	1 n = 32	2 n = 41	3 n = 70	4 n = 74	5 n = 48	6 n = 55	7 n = 38	8 n = 6	9 n = 1	≥ 10 n = 0
AHRE < 6 min n = 269	0% 0/9	0% 0/25	4% 1/25	0% 0/49	3.3% 2/60	15.2% 5/33	12.1% 4/33	25% 8/32	33.3% 1/3	0% 0/0	0% 0/0
AHRE ≥ 6 min n = 107	0% 0/2	0% 0/7	0% 0/16	4.8% 1/21	50% 7/14	33.3% 5/15	27.3% 6/22	83.3% 5/6	33.3% 1/3	0% 0/1	0% 0/0

## Discussion

### The main findings of our study

The R_2_CHA_2_DS_2_-VASc score, and AHRE ≥ 6 min are significantly and independently associated with MACE in patients who with dual-chamber PPMs and no prior AF. The optimal cutoff value for the R_2_CHA_2_DS_2_-VASc score was ≥ 5 for predicting MACE. The other three risk score had acceptable discriminating ability to predict MACE and R_2_CHA_2_DS_2_-VASc score had numerically higher AUC values than others without statistically significant differences. Combination of AHRE ≥ 6 min and R_2_CHA_2_DS_2_-VASc score had an excellent and a better discriminatory power than R_2_CHA_2_DS_2_-VASc score alone or AHRE to predict MACE occurrence. The patients with R_2_CHA_2_DS_2_-VASc scores ≥ 5 and detected AHRE ≥ 6 min had the highest MACE rate (36.2%) at a median follow-up of 32 months. The other three risk scores in combination of AHRE ≥ 6 min also had significantly better discrimination ability to predict MACE. The AUC values were higher in R_2_CHA_2_DS_2_-VASc score and CHA_2_DS_2_-VASc score plus AHRE ≥ 6 min numerically. The results illustrated that when AHRE ≥ 6 min is added to the above four scores, the predictive ability would be enhanced significantly. These findings suggest that further risk assessment with these scores, can further identify the highest risk for MACE in patients with AHRE ≥ 6 min, which may allow early management and prevent MACE.

### The performance of R_2_CHA_2_DS_2_-VASc score as compared to previous studies

The recent studies have shown that R_2_CHA_2_DS_2_-VASc score have better predictive value for long-term outcomes in different groups of patients, including those with high cardiovascular risk, chest pain, and ACS^12–14,17^. In those studies, the reported AUC values of ROC analysis were all above 0.7 for the R_2_CHA_2_DS_2_-VASc score, indicating acceptable discrimination power. The optimal cutoff values for predicting clinical outcomes in those studies were set at ≥ 4 for the R_2_CHA_2_DS_2_-VASc score, and each 1-point increase in the R_2_CHA_2_DS_2_-VASc score was associated with a 31–53% increase in the risk of clinical outcomes^13,14,17^. In our study, the cutoff value was different and set ≥ 5 and each 1-point increase in the R_2_CHA_2_DS_2_-VASc score was associated with a 48% increase in the risk of MACE in patients with dual-chamber PPM, which was comparable to the previous studies in different sets of patients. The area under ROC curve of R_2_CHA_2_DS_2_-VASc score to predict MACE in our study is numerically higher than the reported values^12–14,17^.

### The relationship between AHRE and thrombo-embolism events and possible mechanisms

The rate of AHRE in our study was 28.5%, consistent with previous reported rates (10–30%). In spite of the fact that patients with detected AHRE are at significantly increased risk for stroke, the temporal relationship between device detected AHRE and stroke has not been well established^25–27^. It is possible that AHRE are simply a marker of a population at risk for cardio-embolic events^28^. Miyazawa et al. also demonstrated that longer AHREs more frequently occurred in patients at higher risk of thromboembolism (CHADS2 score ≥ 3)^29^. Pastori et al.^30^ reported that inflammatory markers (high CRP and leukocyte count), are factors in association with AHRE. The mechanism of stroke in patients with implantable devices and detected AHRE may be related to the presence of atherosclerotic risk factors, arterial plaque rupture, inflammation, other than cardio-embolism^27,28^. ^5^. The proposed mechanisms of MACE in patients with AF were systemic inflammation with prothrombotic state; concomitant presence of classic atherosclerotic risk factors including hypertension, diabetes and dyslipidemia associated with platelet activation; and episodes of high ventricular rates leading to supply and demand mismatch and subsequent type 2 MI^5,30^. The increase in cardiovascular risk in patients with AHRE is in line with patients with AF, strengthening the hypothesis that AHRE, also called subclinical AF, and clinical AF are a clinical continuum^7,8^. Thus, the mechanisms of MACE in patients with AF possibly play an important role in patients with AHRE. Previous study^7^ demonstrated that patients with PPM and multiple atherosclerotic risk factors (CHA_2_DS_2_-VASc score > 2), device-detected AHRE can further predict the cardiovascular death and all-cause mortality. Of note, we found that combining R_2_CHA_2_DS_2_-VASc score, consisting of more atherosclerotic risk factors, with AHRE ≥ 6 min as a risk assessment model can further stratify MACE risk (Figs. 3 and 4). The ROC-AUC analysis confirmed the significantly a better predictive value. To the best of our knowledge, the current study is the first to demonstrate that the R_2_CHA_2_DS_2_-VASc score and other three scores combined with AHRE ≥ 6 min have discriminatory power to predict MACE occurrence and the AUC values are statistically higher than the original four risk scores, respectively, in patients with dual-chamber PPM and no prior AF. Kaplan et al.^31^ had shown that there was an interaction between device-detected AF burden and CHA_2_DS_2_-VASc score. The stroke and systemic embolic rates increase across 1%/y in patients with CHA_2_DS_2_-VASc score of 3–4 with > 6 min of AF burden and those with CHA_2_DS_2_-VASc score of 2 with > 23.5 h of AF burden. The results of the study^31^ and ours emphasized the prognostic importance (MACE and stroke) of clinically relevant AHRE in these special population with continuous arrhythmia burden monitoring.

### Current evidence and ongoing trials

Previous study demonstrated the CHADS_2_ and CHA_2_DS_2_-VASc risk scores with integration of AF presence/duration/burden have the potential to improve stroke risk stratification in patients with PPM^32^. Considering stroke prevention, the 2017 European Heart Rhythm Association (EHRA) consensus recommends the consideration of oral anticoagulation (OAC) use for patients with subclinical AF burden > 5.5 h and CHA_2_DS_2_-VASc score ≥ 2. While in 2020 ESC guideline, consideration of OAC use is recommended in patients with subclinical AF burden > 24 h and CHA_2_DS_2_-VASc score ≥ 2 in male and ≥ 3 in female^1^. On the other hand, MACE prevention in patients with device detected AHRE still lacks evidence. A observational cohort study from Danish health care registries reported that direct oral anticoagulation (DOAC) were all associated with a significant risk reduction of MI compared with vitamin K antagonist in patients with non-valvular AF^33^. Two ongoing trial (ARTESiA and NOAH-AFNET 6) will deal with the unmet needs concerning the benefit of apixaban and edoxaban, respectively, for stroke, systemic embolism, or cardiovascular death, as compared with aspirin in patients with AHRE ≥ 6 min^34,35^. The ARTESiA trial will enroll 4000 high-risk (CHA_2_DS_2_-VASc score ≥ 3) patients with CIEDs and at least one AHRE ≥ 6 min. The NOAH-AFNET 6 will recruit 3400 patients aged > 65 years, with one additional CHA_2_DS_2_-VASc factor and CIED-detected AHRE ≥ 6 min. These two trials have the potential to inform future guideline on the management of patients with device detected AHRE to prevent thromboembolism. It is noteworthy that the occurrence of myocardial infarction, acute coronary syndrome or cardiovascular death is secondary outcome rather than primary outcome in the two trials. Therefore, if the trials have positive results for thromboprophylaxis in these patients, further large studies are still needed to investigate whether patients with AHRE have net benefit from use of DOAC to prevent MACE occurrence.

## Limitations

This study has some limitations. First, this was a single-center, retrospective, observational study that enrolled a relatively small number of patients with dual-chamber PPM and no prior AF in a hospital-based setting. Probably due to small number of patients, there was just numerically but not statistically significantly better discriminatory power of R_2_CHA_2_DS_2_-VASc score than other scores to predict MACE. Given the observational design, the cause-effect relationship among the R_2_CHA_2_DS_2_-VASc score, other risk scores, AHRE and MACE could not be determined. Additionally, confounding factors can’t be ruled out. Second, all patients were Taiwanese people. Therefore, the results may not be applicable to other populations. Further prospective and multicenter studies are needed to validate the results of our study. Third, 41.5% of the patients used Biotronik pacemakers, which only recorded an AHRE when heart rate > 200 bpm. This may underestimate the number of AHREs, as patients with a heart rate of 175–200 bpm wouldn’t be recorded as possible AHRE by the devices. Fourth, the severity of common cardiovascular comorbidity factors (e.g., HbA1c level in diabetic patients, blood pressure in hypertensive patients and lipid profile in dyslipidemia patients) would have an impact on all-cause mortality and were not reported in our study.

## Conclusions

In patients with dual-chamber PPM and no prior AF, device-detected AHRE are associated with higher risk for MACE. R_2_CHA_2_DS_2_-VASc score with the cut-off value of 5 can be used to predict MACE in patients with dual chamber PPM and no prior AF. Combination of AHRE and R_2_CHA_2_DS_2_-VASc score to predict MACE has an acceptable discriminatory power, which is comparable to other three risk scores. When adding AHRE ≥ 6 min to the four risk scores, all demonstrate significantly better and comparable discriminatory power to predict MACE. Our study suggests the significant prognostic importance of risk assessment, e.g., R_2_CHA_2_DS_2_-VASc score, in PPM patients with detected AHRE ≥ 6 min. In dual chamber PPM patients with high risk in R_2_CHA_2_DS_2_-VASc score, and with device-detected AHRE ≥ 6 min plus intermediate risk in R_2_CHA_2_DS_2_-VASc score, early management may be warranted to prevent MACE occurrence. Optimization of thrombo-embolism prevention strategy with DOACs in patients AHRE is under investigation. Large cohort studies are in need to address whether DOACs can reduce cardiovascular mortality in patients with AHRE in the future.

## Data Availability

The datasets generated and analyzed during the current study are not publicly available due to privacy restriction but can be made available from the corresponding author on reasonable request.

## Abbreviations

AF: Atrial fibrillation
AHRE: Atrial high-rate episodes
AMI: Acute myocardial infarction
CIEDs: Cardiac implantable electronic devices; disease
eGFR: Estimated glomerular filtration rate
TIA: Transient ischemic attacks
